# *rpoB* mutations and their association with rifampicin resistance in clinical *Staphylococcus epidermidis*

**DOI:** 10.1093/jac/dkaf035

**Published:** 2025-02-06

**Authors:** Sofie Marie Edslev, Mia Aarris, Karen Leth Nielsen, Frederik B Hertz, Thor Bech Johannesen, Camille Kolenda, Frederic Laurent, Emeli Månsson, Bo Söderquist, Marc Stegger

**Affiliations:** Department of Sequencing and Bioinformatics, Statens Serum Institut, Copenhagen, Denmark; European Society of Clinical Microbiology and Infectious Diseases (ESCMID) Study Group for Staphylococci and Staphylococcal Diseases (ESGS), Basel, Switzerland; Department of Sequencing and Bioinformatics, Statens Serum Institut, Copenhagen, Denmark; European Society of Clinical Microbiology and Infectious Diseases (ESCMID) Study Group for Staphylococci and Staphylococcal Diseases (ESGS), Basel, Switzerland; Department of Clinical Microbiology, Copenhagen University Hospital - Rigshospitalet, Copenhagen, Denmark; Department of Clinical Microbiology, Copenhagen University Hospital - Rigshospitalet, Copenhagen, Denmark; Department of Clinical Microbiology, Copenhagen University Hospital - Rigshospitalet, Copenhagen, Denmark; Department of Immunology & Microbiology, University of Copenhagen, Copenhagen, Denmark; Department of Sequencing and Bioinformatics, Statens Serum Institut, Copenhagen, Denmark; European Society of Clinical Microbiology and Infectious Diseases (ESCMID) Study Group for Staphylococci and Staphylococcal Diseases (ESGS), Basel, Switzerland; European Society of Clinical Microbiology and Infectious Diseases (ESCMID) Study Group for Staphylococci and Staphylococcal Diseases (ESGS), Basel, Switzerland; Service de Bactériologie, Centre National de Référence des Staphylocoques, Institut des Agents Infectieux, Hospices Civils de Lyon, Lyon, France; Equipe StaPath, CIRI, Centre International de Recherche en Infectiologie, Université de Lyon, Lyon, France; European Society of Clinical Microbiology and Infectious Diseases (ESCMID) Study Group for Staphylococci and Staphylococcal Diseases (ESGS), Basel, Switzerland; Service de Bactériologie, Centre National de Référence des Staphylocoques, Institut des Agents Infectieux, Hospices Civils de Lyon, Lyon, France; Equipe StaPath, CIRI, Centre International de Recherche en Infectiologie, Université de Lyon, Lyon, France; European Society of Clinical Microbiology and Infectious Diseases (ESCMID) Study Group for Staphylococci and Staphylococcal Diseases (ESGS), Basel, Switzerland; Region Västmanland—Uppsala University, Centre for Clinical Research, Västmanland Hospital Västerås, Västerås, Sweden; Department of Medical Sciences, Uppsala University, Uppsala, Sweden; European Society of Clinical Microbiology and Infectious Diseases (ESCMID) Study Group for Staphylococci and Staphylococcal Diseases (ESGS), Basel, Switzerland; School of Medical Sciences, Faculty of Medicine and Health, Örebro University, Örebro, Sweden; Department of Laboratory Medicine, Clinical Microbiology, Faculty of Medicine and Health, Örebro University, Örebro, Sweden; Department of Sequencing and Bioinformatics, Statens Serum Institut, Copenhagen, Denmark; European Society of Clinical Microbiology and Infectious Diseases (ESCMID) Study Group for Staphylococci and Staphylococcal Diseases (ESGS), Basel, Switzerland; Antimicrobial Resistance and Infectious Diseases Laboratory, Harry Butler Institute, Murdoch University, Murdoch, WA, Australia

## Abstract

**Background:**

*Staphylococcus epidermidis* is a ubiquitous member of the healthy skin and mucous microbiota but is also an opportunistic pathogen responsible for various infections, often treated with antibiotics like rifampicin. Resistance to rifampicin in *S. epidermidis* arises primarily through nonsynonymous mutations in the *rpoB* gene.

**Objectives:**

To investigate the prevalence of *rpoB* mutations and their association with phenotypic rifampicin resistance in clinical *S. epidermidis* isolates from Denmark, France, and Sweden.

**Methods:**

All clinical isolates (N = 942) were whole-genome sequenced to identify mutations in *rpoB* and subsequently linked to phenotypic rifampicin resistance based on antimicrobial susceptibility testing.

**Results:**

A total of 64 (6.8%) isolates were resistant to rifampicin. They carried all mutational changes in the rifampicin resistance-determining region (RRDR). Among 12 identified nonsynonymous mutations, 11 were exclusively observed in resistant strains, including novel mutations not previously described in *S. epidermidis*.

**Conclusions:**

This study highlights the diverse genetic variants of *rpoB* associated with rifampicin resistance in clinical *S. epidermidis* isolates, including novel mutations. The strong correlation between mutational changes in RRDR and phenotypic resistance reinforces the role of *rpoB* mutations as a primary mechanism of resistance in clinical isolates.

## Introduction

*Staphylococcus epidermidis* is a common cause of infections associated with indwelling medical devices and prosthetic joints.^1,2^ Biofilm formation is crucial in establishing these infections, as it protects bacteria from the host immune response and antibiotic treatment, resulting in challenging clinical scenarios. Rifampicin is a bactericidal antibiotic used in combination therapy to treat implant-associated staphylococcal infections as it can penetrate biofilms and target slow-growing and intracellular bacteria effectively.^1^ Rifampicin inhibits RNA synthesis by binding to the *rpoB*-encoded β subunit of the RNA polymerase. Resistance results from mutations within specific regions of the *rpoB* gene, named the rifampicin resistance-determining region (RRDR), which comprises the transcription cleft and active site of the enzyme.^3^ In *S. epidermidis*, RRDR consists of three clusters: Cluster I (1378–1476, aa 460–492), Cluster II (1549–1590, aa 517–530) and Cluster III (1921–1941, aa 641–647).

Rifampicin resistance in *S. epidermidis* has been documented primarily in prosthetic joint infections (PJIs), with reported prevalence ranging from 7% to 8% to as high as 39%.^2,4^ However, data on the overall prevalence of rifampicin resistance in clinical *S. epidermidis* strains remains limited due to the lack of routine surveillance. Reports from reference laboratories suggest significant local variation in rifampicin resistance rates, with estimates ranging from 3% to 20% depending on the institution and time period.^5^

Mutations in the *rpoB* gene are well characterized in *Staphylococcus aureus*, not only for their role in conferring resistance to rifampicin but also for their association with decreased susceptibility to last-resort antibiotics like vancomycin and daptomycin.^6^ In contrast, sparse knowledge about *rpoB* mutations in *S. epidermidis* is available. Among the few identified are H481Y associated with high-level rifampicin resistance, and the double mutation D471E/I527M that also reduce susceptibility to vancomycin and teicoplanin.^5,7^ The D471E/I527M variant is the most prevalent cause of rifampicin resistance within the hospital-associated lineages ST2, ST5, and ST23 accounting for ∼85% of resistant cases.^5^ This study examines the prevalence of *rpoB* mutations across clinical isolates of *S. epidermidis* and their association to rifampicin resistance while reporting previously unidentified *rpoB* mutations within *S. epidermidi*s from Denmark, France and Sweden.

## Materials and methods

### Isolate collection

Clinical *S. epidermidis* isolates originating from three countries were included in the study: First, 53 isolates from PJI cases in France (the Lyon University Hospital) collected during 2015–20; second, 138 isolates from PJI cases in Sweden (Hospitals in Region Örebro County and Region Östergötland) collected during 2007–16;^7^ and third, a collection of 751 isolates from various human infections in Denmark received at a tertiary hospital (Rigshospitalet, Copenhagen) during 2022–24.

One isolate from each patient was included in the study. Rifampicin susceptibility was assessed for Swedish and Danish isolates using the disk diffusion method according to EUCAST guidelines^8^ (R < 30 mm inhibition zones). For French isolates, resistance was assessed based on MICs using VITEK2 (bioMérieux Inc.), where resistance was defined by a MIC >0.5 mg/L.

### Genome sequencing and bioinformatics

Whole-genome sequencing of all the isolates was performed using Illumina sequencing technology (Illumina Inc., San Diego, CA, USA).

Sequences were assembled using SPAdes v.3.13.1 with default settings^9^ and MLSTs identified using mlst v.2.11 (https://github.com/tseemann/mlst). A basic local alignment search tool (BLASTN) analysis of the assembled genomes against a *rpoB* sequence (GenBank accession no. AE015929, locus ID SE_0306) was performed in Geneious Prime 2024.0.7 (https://www.geneious.com), followed by a manual screening for all nonsynonymous mutations. Results were validated with read mapping using ARIBA v. 2.14.6.^10^ Summary statistics and plots of the mutation prevalence were performed in R v.4.2.1 using the ggplot2 v.3.4.1 package. Protein structures (UniProt RpoB ID: A0A0E1VH12) were visualized using PyMOL v.3.0.4 based on the *S. epidermidis* RpoB sequence, and amino acid substitutions were generated using PyMOL’s Mutagenesis Wizard tool.^11^

## Results

A total of 942 *S. epidermidis* isolates from human infections, representing a diverse genetic background (>125 different STs), were included in the study. Sixty-four of the isolates (6.8%) were phenotypically resistant to rifampicin, and they all carried nonsynonymous mutations in the RRDR of *rpoB*. The majority (81%) of the resistant isolates belonged to hospital-adapted lineages, namely ST2 (N = 24), ST23 (N = 17), and ST5 (N = 11).

**Figure 1.**
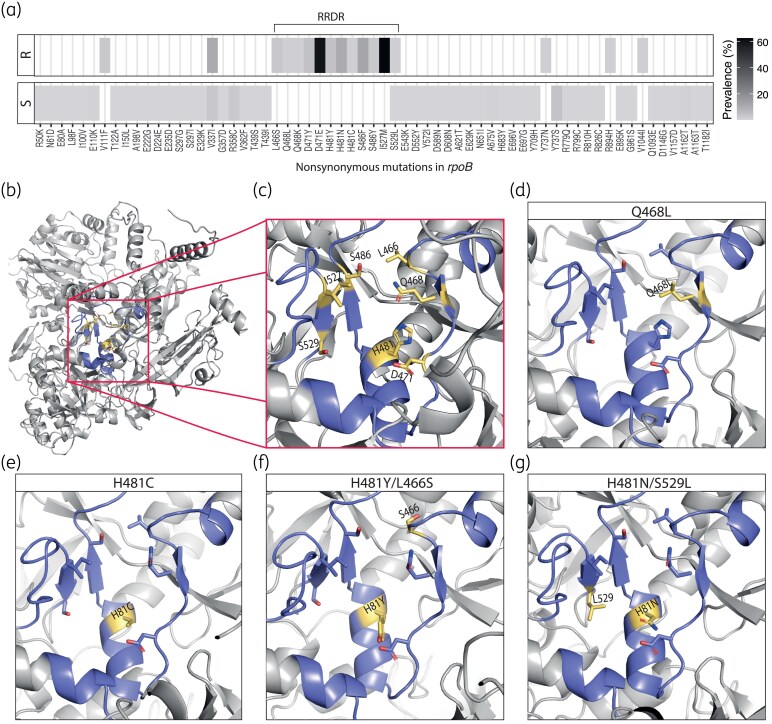
Distribution of mutations in *rpoB* and structural depiction of the RpoB active site. (a) Proportional prevalence of nonsynonymous mutations in *rpoB* in *S. epidermidis* isolates that were susceptible (S), and resistant (R) to rifampicin. (b) Crystal structure of the 1183 aa RpoB and (c) close-up of the active site, showing the RRDR and the seven amino acids previously linked to rifampicin resistance in *S. epidermidis* or *S. aureus*. (d–g) Structural models displaying active site substitutions: (d) Q468L, (e) H481C, (f) H481Y/L466S and (g) H481N/S529L. The visualizations of the protein structure were made with PyMOL v.3.0.4.

Twelve different nonsynonymous mutations within Cluster I and Cluster II of RRDR were identified (Table 1), of which 11 were exclusive to resistant isolates and absent from all susceptible isolates (N = 878). The exception was S529L which was detected in a single rifampicin-susceptible isolate [Figure 1a, Table S1 (available as Supplementary data at *JAC* Online)]. No mutations within Cluster III of the RRDR were identified in any of the isolates.

**Table 1.** Mutations in rifampicin resistance-determining regions of the *rpoB* gene in rifampicin-resistant *S. epidermidis*

Amino acid substitution	Single nucleotide polymorphism	Prevalence, N (%)	Lineages	References	
				*S. epidermidis*	*S. aureus*
Q468K^a^	C1402A	1 (1.6%)	ST5 (N = 1)	Wi *et al.*^1^	—
Q468L^b^	A1403T	1 (1.6%)	Other ST (N = 1)	This study	Matsuo *et al.*^12^
D471Y^b,c^	G1411T	3 (4.7%)	ST2 (N = 1), ST5 (N = 1), Other ST (N = 1)	This study	Lee *et al.*^4^
H481C	C1441T and A1442G	1 (1.6%)	ST215 (N = 1)	This study	—
H481N^a^	C1441A	3 (4.7%)	ST434 (N = 2), Other ST (N = 1)	Gostev *et al.*^13^ Widerström *et al.*^14^	—
H481Y^a^	C1441T	2 (3.1%)	ST5 (N = 2)	Wi *et al.*^1^ Hellmark *et al*.^2^	—
S486F^a^	C1457T	8 (12.5%)	ST5 (N = 4), ST2 (N = 3), ST215 (N = 1)	Albano *et al.*^15^	—
S486Y^a^	C1457A	2 (3.1%)	ST2 (N = 1), ST5 (N = 1)	Widerström *et al.*^14^	—
H481N/L466S^b^	C1441A and T1397C	1 (1.6%)	ST215 (N = 1)	This study	Guérillot *et al.*^6^ Guo *et al.*^16^
H481Y/L466S	C1441T and T1397C	1 (1.6%)	ST5 (N = 1)	This study	—
D471E/I527M^a^	C1413A and A1581G	38 (59.4%)	ST2 (N = 19), ST23 (N = 17), ST87 (N = 1), ST1083 (N = 1)	Lee *et al.*^5^ Petti *et al.*^17^	—
H481N/I527M^a^	C1441A and A1581G	2 (3.1%)	ST434 (N = 2)	Petti *et al.*^17^	—
H481N/S529L^b^	C1441A and A1581G	1 (1.6%)	ST5 (N = 1)	This study	Guérillot *et al.*^6^

The table includes all nonsynonymous mutations identified within the RRDR of the *rpoB* gene. The proportional prevalence was calculated based on the total number of rifampicin-resistant isolates (N = 64). Isolates with a novel MLST profile was defined as *Other ST*.

^a^Previously described in *S. epidermidis*.

^b^Previously described in *S. aureus*.

^c^Previously described in *S. epidermidis* but only in combination with other RRDR mutations.

Two of the identified mutations in RRDR, Q468L and H481C (Figure 1d and e), have not previously been identified in *S. epidermidis*. Q468L was detected in an isolate from Denmark of novel ST (single locus variant of ST110) and was the only mutation in *rpoB*. The H481C variant was detected as the only mutation in a ST215 isolate from Sweden besides a fixed mutation outside RRDR, V337I present in all ST215 isolates. Rifampicin susceptibility testing revealed no or small (11 mm) zones of inhibition for the two isolates, respectively (Figure S1).

The most prevalent cause of resistance was the co-carriage of the D471E/I527M mutations, identified in 38 isolates (59%), primarily, but not exclusively, in ST2 and ST23 (Table 1). Five additional isolates harboured multiple mutations in RRDR. Specifically, two isolates co-carried the H481Y/L466S or H481N/L466S mutations that were associated with high-level resistance (no zones of inhibition) (Figure 1f, Figure S1). In addition, two ST434 isolates co-carried H481N/I527M, and one ST5 isolate co-carried H481N/S529L (Figure 1g).

Nonsynonymous mutations outside the RRDR of *rpoB* were detected in 153 isolates, including both susceptible and resistant isolates, but were all found in low prevalence (Figure 1a, Table S1). Four resistant isolates had mutations outside the RRDR, which were only observed in resistant strains (V111F, Y737N, R894H, V1044I); however, these isolates all carried mutations within the RRDR known to cause resistance in either *S. epidermidis* or *S. aureus*.

## Discussion

This study identified multiple mutations in the *rpoB* gene linked to phenotypic rifampicin resistance in a large collection of clinical *S. epidermidis* isolates. Our findings revealed rifampicin resistance consistently correlated (100%) with mutations in the RRDR of the *rpoB* gene, supporting that *rpoB* mutations is the primary mechanism of rifampicin resistance in clinical *S. epidermidis*. The double mutation D471E/I527M was the most prevalent cause of resistance as expected.^5^ This study further uncovered a range of mutations contributing to resistance, including Q468L and H481C, which has not been previously reported in *S. epidermidis*. These mutations were found exclusively in isolates with high-level resistance, strongly suggesting their significant contribution to rifampicin resistance. Similar mutations (Q468K, H481N/Y) have previously been associated with rifampicin resistance either in *S. aureus* or *S. epidermidis.*^1,2,13,17^ The D471Y mutation has previously been reported in combination with other RRDR mutations;^4,17^ however, to the best of our knowledge, this study is the first to demonstrate that D471Y alone can confer rifampicin resistance in *S. epidermidis*.

Co-carriage of the mutations H481Y/L466S, H481N/L466S and H481N/S529L was identified in a single isolate. These double mutations, although widespread in *S. aureus*,^6,16,18,19^ has not previously been observed in *S. epidermidis*, suggesting some species-specific differences. For H481N, combinations of RRDR mutations seem to confer greater resistance than single mutations alone (Figure S1). Both L466S and S529L have been linked to low-level rifampicin resistance in *S. aureus*,^6,18,20^ while their combination with H481N is associated with high-level resistance^16,18^ and reduced susceptibility to vancomycin and daptomycin.^6^ The identification of S529L in a susceptible isolate suggests that S529L alone does not confer resistance in *S. epidermidis* unlike in *S. aureus*.

Whether the L466S variant alone can mediate low-level resistance in *S. epidermidis* is uncertain, as this mutation was only detected in combination with other mutations known to cause resistance.

A key strength of this study is the inclusion of a large number of clinical isolates from various specimen types across multiple countries, representing diverse clonal backgrounds and geographical origin. With the exception of one study,^5^ previous research on *rpoB* mutations in *S. epidermidis* has relied on significantly smaller collections of isolates,^2,4,14,17^ limiting the ability to detect low-prevalence mutations and accurately estimate their overall prevalence.

A limitation of studying clinical isolates is the varying genetic backgrounds of the isolates, which may influence resistance beyond the presence of *rpoB* mutations. Developing genotypically identical clones for each *rpoB* variant *in vitro* could clarify the role of specific mutations. Additionally, determining the MIC for all isolates would have been beneficial to better quantify the impact of individual RRDR mutations more precisely. Lastly, the inclusion of information on infection types, as well as data on treatment regimens and clinical outcomes, would have added further depth to our findings. Unfortunately, this information was not available for these isolates.

In conclusion, this study underscores the diverse *rpoB* genetic variants associated with rifampicin resistance in clinical *S. epidermidis* isolates, including novel *rpoB* mutations. Given the critical role of rifampicin in combination therapy and the potential impact of *rpoB* mutations on vancomycin and daptomycin susceptibility in other staphylococci, understanding resistance mechanisms and monitoring their prevalence is essential for optimized treatment strategies.

## Supplementary Material

dkaf035_Supplementary_Data

## Data Availability

The *rpoB* gene sequences from the isolates and a sample data file are available on GitHub (https://github.com/ssi-dk/S_epidermidis_rpoB_point_mutations/tree/main). Other data can become available for research upon requests to the corresponding author, and within the framework of the Danish data protection legislation and any required permission from the authorities.

## Supplementary data

Figure S1 and Table S1 are available as Supplementary data at *JAC* Online.
