# Supplementary material for: rpoB mutations and their association with rifampicin resistance in clinical Staphylococcus epidermidis
Source: J Antimicrob Chemother. 2025 Feb 6;80(4):1067–71. doi: 10.1093/jac/dkaf035 (PMC11962384; doi:10.1093/jac/dkaf035)
Supplement: dkaf035_Supplementary_Data [file dkaf035_supplementary_data.docx]

**Supplementary figure 1. Zones of inhibition in relation to *rpoB* mutations in rifampicin-resistant isolates.** The association between the presence of nonsynonymous mutations in the rifampin resistance-determining region (RRDR) of the *rpoB* gene and the zones of inhibition measured by disk diffusion. A 6 mm inhibition zone is considered equivalent to no inhibition zone. The data include rifampin-resistant isolates collected in Sweden and Denmark. Information on inhibition zones was not available for the French isolates.

**Supplemental table 1. Mutations in the *rpoB* gene in rifampicin-resistant and rifampicin-susceptible *S. epidermidis***

| **aa substitutions** | **Resistant isolates (N=64)** | | **Susceptible isolates (N=878)** | |
| --- | --- | --- | --- | --- |
|  | **Prevalence N (%)** | **MLST** | **Prevalence**  **N (%)** | **MLST** |
| R50K | 0 |  | 2 (0.3%) | Other ST (N=2) |
| N61D | 0 |  | 1 (0.1%) | ST327 (N=1) |
| E80A | 0 |  | 1 (0.1%) | ST297 (N=1) |
| L98F | 0 |  | 1 (0.1%) | ST66 (N=1) |
| I100V | 0 |  | 2 (0.3%) | ST170 (N=2) |
| E110K | 0 |  | 6 (0.7%) | ST378 (N=4), ST520 (N=1),Other ST (N=1) |
| V111F | 2 (3%) | ST5 (N=2) | 0 |  |
| T122A | 0 |  | 1 (0.1%) | ST5 (N=1) |
| I150L | 0 |  | 1 (0.1%) | ST200 (N=1) |
| A198V | 0 |  | 1 (0.1%) | ST73 (N=1) |
| E222G | 0 |  | 1 (0.1%) | ST5 (N=1) |
| D224E | 0 |  | 1 (0.1%) | ST87 (N=1) |
| E235D | 0 |  | 1 (0.1%) | ST218 (N=1) |
| S297G | 0 |  | 1 (0.1%) | ST35 (N=1) |
| S297I | 0 |  | 1 (0.1%) | ST17 (N=1) |
| E329K | 0 |  | 1 (0.1%) | ST200 (N=1) |
| V337I | 7 (11%) | ST215 (N=3), ST434 (N=4) | 27 (3%) | ST215 (N=25), ST730 (N=1),Other ST (N=1) |
| G357D | 0 |  | 1 (0.1%) | ST217 (N=1) |
| R358C | 0 |  | 26 (3%) | ST230 (N=1), ST48 (N=11),ST54 (N=4),ST558 (N=2),ST89 (N=1),ST987 (N=1),ST993 (N=1),Other ST (N=5) |
| V362F | 0 |  | 1 (0.1%) | ST2 (N=1) |
| T439S | 0 |  | 1 (0.1%) | ST48 (N=1) |
| T439I | 0 |  | 1 (0.1%) | Other ST (N=1) |
| L466S | 2 (3%) | ST5 (N=1),ST215 (N=1) | 0 |  |
| Q468L | 1 (2%) | Other ST (N=1) | 0 |  |
| Q468K | 1 (2%) | ST5 (N=1) | 0 |  |
| D471Y | 3 (5%) | ST2 (N=1), ST5 (N=1),Other ST (N=1) | 0 |  |
| D471E | 38 (59%) | ST1083 (N=1),ST2 (N=19),ST23 (N=17),ST87 (N=1) | 0 |  |
| H481Y | 3 (5%) | ST5 (N=3), | 0 |  |
| H481N | 7 (11%) | ST215 (N=1), ST434 (N=4), ST5 (N=1), Other ST (N=1) | 0 |  |
| H481C | 1 (2%) | ST215 (N=1) | 0 |  |
| S486F | 8 (13%) | ST2 (N=3), ST215 (N=1), ST5 (N=4) | 0 |  |
| S486Y | 2 (3%) | ST2 (N=1), ST5 (N=1) | 0 |  |
| I527M | 40 (63%) | ST1083 (N=1), ST2 (N=19), ST23 (N=17), ST434 (N=2), ST87 (N=1) | 0 |  |
| S529L | 1 (2%) | ST5 (N=1) | 1 (0.1%) | Other ST (N=1) |
| E543K | 0 |  | 1 (0.1%) | Other ST (N=1) |
| D552Y | 0 |  | 2 (0.3%) | ST2 (N=2) |
| Y572I | 0 |  | 1 (0.1%) | ST88 (N=1) |
| D589N | 0 |  | 4 (0.5%) | ST5 (N=4) |
| D608N | 0 |  | 1 (0.1%) | ST7 (N=1) |
| A621T | 0 |  | 1 (0.1%) | ST640 (N=1) |
| E629K | 0 |  | 1 (0.1%) | ST215 (N=1) |
| N651I | 0 |  | 1 (0.1%) | ST101 (N=1) |
| A675V | 0 |  | 6 (0.7%) | ST378 (N=4), ST520 (N=1), Other ST (N=1) |
| H683Y | 0 |  | 1 (0.1%) | ST215 (N=1) |
| E696V | 0 |  | 1 (0.1%) | ST5 (N=1) |
| E697G | 0 |  | 1 (0.1%) | ST452 (N=1) |
| Y709H | 0 |  | 3 (0.3%) | ST60 (N=2), Other ST (N=1) |
| Y737N | 1 (2%) | ST87 (N=1) | 0 |  |
| Y737S | 1 (2%) | Other ST (N=1) | 39 (4%) | ST1012 (N=1), ST1044 (N=2),ST19 (N=7),ST264 (N=1),ST5 (N=1),ST640 (N=15),ST723 (N=4),ST89 (N=5),Other ST (N=3) |
| R779Q | 0 |  | 1 (0.1%) | Other ST (N=1) |
| R799C | 0 |  | 1 (0.1%) | ST21 (N=1) |
| R810H | 0 |  | 1 (0.1%) | Other ST (N=1) |
| R826C | 0 |  | 1 (0.1%) | ST886 (N=1) |
| R894H | 1 (2%) | ST434 (N=1) | 0 |  |
| E895K | 0 |  | 1 (0.1%) | Other ST (N=1) |
| G961S | 0 |  | 1 (0.1%) | ST173 (N=1) |
| V1044I | 1 (2%) | ST215 (N=1) | 0 |  |
| Q1093E | 0 |  | 1 (0.1%) | ST87 (N=1) |
| D1146G | 0 |  | 1 (0.1%) | ST297 (N=1) |
| V1157D | 0 |  | 2 (0.3%) | ST73 (N=2) |
| A1162T | 0 |  | 6 (0.7%) | ST378 (N=4), ST520 (N=1), Other ST (N=1) |
| A1163T | 0 |  | 4 (0.5%) | ST624 (N=1), Other ST (N=3) |
| T1182I | 0 |  | 1 (0.1%) | ST89 (N=1) |
